# False but phonologically plausible linguistic priors induce cross-linguistic auditory illusions and attenuate electrophysiological markers of surprise

**DOI:** 10.1162/IMAG.a.1178

**Published:** 2026-03-26

**Authors:** Enrico Giraldi, Mirco Frosolone, Andrew W. Corcoran, Laura Barca, Alessandro Corsini, Francesco Donnarumma, Alessandro D’Ausilio, Giovanni Pezzulo

**Affiliations:** Institute of Cognitive Sciences and Technologies, National Research Council, Rome, Italy; University of Camerino, Camerino, Italy; Monash University, Melbourne, Australia; University of Ferrara, Ferrara, Italy; Center for Translational Neurophysiology of Speech and Communication, Istituto Italiano di Tecnologia, Ferrara, Italy

**Keywords:** auditory illusion, predictive coding, predictive routing, EEG

## Abstract

According to predictive coding theory, perception arises from the interplay between top–down expectations and bottom–up sensory input. In the auditory domain, this interplay can lead to illusory experiences—for instance, when phonetic similarities between languages cause listeners to mishear foreign lyrics as familiar words in their native language. This study investigated how linguistic priors influence auditory perception during song listening. Participants read sentences in English or Italian before hearing English song excerpts, then rated how well the lyrics matched the text. Crucially, some of the Italian sentences were phonologically similar to the English lyrics, resulting in a cross-linguistic auditory illusion (“Illusory Trials”) in which listeners reported the content of the lyrics matched that of the Italian text. EEG analysis showed that two neural markers of surprise—P200 amplitude and gamma-band activity—were heightened while listening to songs that were not perceived to match the preceding text, reflecting the violation of expectations. In contrast, these markers were attenuated in illusory trials, suggesting reduced prediction error despite the lack of semantic match. Time–frequency analysis revealed an early gamma response followed by beta-band dominance in the illusory condition, consistent with initial sensory mismatch followed by top–down assimilation. Together, these findings show that phonologically plausible but false linguistic priors can induce auditory illusions and modulate predictive processing during song perception.

## 1. Introduction

It is common wisdom that we often see what we expect to see and hear what we expect to hear. A growing body of research demonstrates that prior expectations play a dual role: they can facilitate and speed up the recognition of expected stimuli (Bar, 2004), and they can shape the very content of what is perceived, particularly under conditions of ambiguity. For example, expectations can influence the perception of visual motion (Sterzer et al., 2008) or the identification of spoken words (Leonard et al., 2016). Linguistically induced expectations make people aware of otherwise invisible images (Lupyan & Clark, 2015; Lupyan & Ward, 2013).

Notably, prior expectations do not merely bias perception—they can generate illusory percepts in the absence of corresponding sensory input (Aru et al., 2018; Geisler and Kersten, 2002). For instance, auditory hallucinations of speech can be induced using language templates (Alderson-Day et al., 2022) or Pavlovian conditioning paradigms (Powers et al., 2017). Outside of the laboratory, magicians routinely exploit our prior expectations—both implicit and explicit—to produce perceptual illusions. A classic example involves the “vanishing ball” illusion, where viewers perceive a ball to disappear based on their expectations about the trajectory of physical actions (Kuhn & Rensink, 2016).

The influence of prior expectations on conscious experience can be understood within the framework of *predictive coding*, a computational theory of brain function that posits that perception arises from the integration of top–down predictions and bottom–up sensory input (Bastos et al., 2012; Clark, 2013; De Lange et al., 2018; Dimakou et al., 2025; Friston, 2005; Hohwy, 2013; Parr et al., 2022; Pezzulo et al., 2021; Rao & Ballard, 1999). According to this view, the brain continuously generates predictions based on prior knowledge and contextual cues, and compares these predictions with actual sensory signals. Discrepancies—termed prediction errors—are then used to update internal models to reduce future error. Perceptual illusions can be conceptualized within this framework, by considering that they result from (Bayes-optimal) integration of priors and sensory evidence—and when the latter is ambiguous, perception is more strongly biased toward one’s prior expectations (Brown & Friston, 2012; Brown et al., 2013; Parr et al., 2019).

Predictive coding has been widely investigated in the auditory domain, encompassing both speech and music perception (Bonetti et al., 2022, 2024; Koelsch et al., 2019; Popescu et al., 2024; Vuust & Witek, 2018). Research has focused on two key constructs: the formation of expectations about upcoming auditory input and neural responses to violations of those expectations—that is, prediction errors. Studies have demonstrated that these predictive processes are instantiated throughout the auditory hierarchy, engaging both cortical regions (Heilbron & Chait, 2018; Kok & de Lange, 2015; Summerfield & De Lange, 2014) and subcortical structures (Tabas et al., 2020). Language-based expectations have also been shown to significantly shape auditory processing. Linguistic priors—derived from phonetic, syntactic, or semantic information—can enhance comprehension of noisy or ambiguous speech (Muncke et al., 2022). Previous behavioral studies have shown that linguistic context can bias auditory perception, leading listeners to misidentify words that are acoustically similar to those expected from the context. This phenomenon, known as *false hearing*, reflects the dominance of top–down semantic expectations over bottom–up acoustic input (Rogers, 2017; Rogers & Wingfield, 2015; Rogers et al., 2012). Recent work has further demonstrated that false hearing and false memory share a common underlying mechanism, reflecting failures of cognitive control that allow prior semantic expectations to override incoming sensory evidence (Failes et al., 2020). Building on this idea, Van Os et al. (2021) proposed that mishearing arises as a rational consequence of Bayesian language comprehension in noisy contexts, when top–down expectations dominate over ambiguous sensory evidence. For instance, predictions based on sentence structure can aid in reconstructing degraded auditory input from brain signals (Corcoran et al., 2023), and semantic congruency between context and stimulus enhances both accuracy and efficiency of auditory perception, as reflected in the modulation of event-related potentials (Bendixen et al., 2009; Kutas & Hillyard, 1980; Shain et al., 2020).

Robust neural signals reflecting both prediction and prediction error have been observed during speech perception (Blank & Davis, 2016; Goldstein, Weiss, et al., 2022; Hockley et al., 2025; Momenian et al., 2024). Early auditory event-related potentials (ERPs), particularly the N1 and P2 components, have been extensively investigated as indices of sensitivity to acoustic–phonological regularities and context-based expectations in speech. The N1 reflects early stages of auditory analysis and has been shown to vary with phonological and lexical predictability in spoken language (Sharma et al., 1997; Yue et al., 2022), often showing reduced amplitude for predicted or repeated stimuli—a phenomenon described as repetition suppression or predictive attenuation (Bendixen et al., 2012). However, evidence for a direct one-to-one mapping between the N1 and prediction-error magnitude remains mixed; we, therefore, interpret N1 modulation primarily as an index of early sensitivity to expectancy and phonological regularities rather than a specific neural correlate of prediction error. The subsequent P2 component has been linked to the perceptual integration of expected phonetic features and to attentional enhancement of salient auditory cues. Training and exposure studies demonstrate that P2 amplitude increases with the consolidation of stable auditory representations (Tremblay et al., 2010, 2014; Tremblay & Kraus, 2002), and is modulated by the degree of perceptual salience in speech and music contexts (Pinheiro et al., 2022). In this framework, P2 provides a complementary marker of early perceptual integration and attentional engagement during the processing of expected versus unexpected linguistic input. Together, modulation of N1 and P2 thus offers a theoretically grounded—yet conservative—window into early predictive processes in speech perception.

A growing number of studies have examined the oscillatory mechanisms underlying predictive coding, suggesting that distinct frequency bands appear to mediate different aspects of predictive processing. In this perspective, high beta rhythms (~20–30 Hz) are implicated in the top–down transmission of predictions from higher-order areas to sensory cortices, potentially stabilizing perceptual representations, whereas low gamma rhythms (~30–50 Hz) are associated with the bottom–up signaling of prediction errors resulting from violations of expected sensory input (Arnal & Giraud, 2012; Bastos et al., 2012; Engel & Fries, 2010; Engel et al., 2001; Friston et al., 2015; Spitzer & Haegens, 2017; Zhang et al., 2019). The *predictive routing* model suggests that the coordinated interplay between these oscillatory bands enables the brain to flexibly integrate expectations with sensory evidence. Specifically, alpha and beta oscillations would inhibit feedforward gamma activity to facilitate the processing of expected stimuli—or conversely allow feedforward gamma activity when stimuli are unexpected (Bastos et al., 2020).

While predictive coding and predictive routing have advanced our understanding of how the brain integrates prior knowledge with sensory input, their role in shaping auditory perception particularly in response to linguistic expectations remains incompletely understood. Specifically, it is still unclear whether and how linguistic priors influence what we perceive when processing complex auditory input. Can linguistic expectations lead to the illusion of hearing words that were not actually spoken? What neural mechanisms underlie the brain’s ability to distinguish between congruent, incongruent, and illusory congruent stimuli shaped by prior linguistic context?

To address these questions, we investigated whether false but phonologically plausible linguistic priors can give rise to cross-linguistic auditory illusions, and how such illusions are manifest behaviorally and neurally. Participants engaged in a task in which they first read an English or Italian sentence and then listened to an English song lyric. Crucially, in some cases, the Italian sentence was phonologically similar to the English lyric, producing the perceptual illusion of hearing Italian words in an English-language song—a phenomenon popularized in Italian pop culture through the comedic radio segment Canzoni Travisate (“misheard songs”). For example, reading the Italian sentence “le galline con le spine” before hearing the English lyric “Like a wheel, gonna spin it” from the AC/DC song Highway to Hell often leads listeners to report hearing the Italian phrase within the English audio. We refer to this as a cross-linguistic auditory *illusion*, as the perceived sentence (in Italian) systematically differs from the actual spoken sentence (in English).

We collected participants’ subjective reports to assess whether they experienced the illusion and recorded their electroencephalogram (EEG) activity to examine differences in predictive processing between the illusory condition and conditions in which the linguistic priors were either veridical or false and phonologically incongruent with the heard lyrics.

We hypothesized that veridical priors would elicit lower prediction error than incongruent priors. Crucially, however, false but phonologically congruent linguistic priors were expected to induce an illusory auditory experience of Italian words—and to elicit reduced prediction error relative to the incongruent condition. We focused our analysis on early event-related potentials (ERPs), which are closely linked to auditory predictive processing. Specifically, we predicted that ERPs associated with surprise—namely N1 and P2 amplitudes—would be enhanced in both the incongruent and illusory conditions relative to the congruent condition, reflecting violations of prior expectations. However, this amplitude increase should be attenuated in the illusory condition compared with the incongruent one, due to partial assimilation of the unexpected input. Additionally, we hypothesized that beta oscillatory activity—associated with predictive engagement—would dominate in the congruent condition, while gamma activity—reflecting prediction error—would initially dominate in both the incongruent and illusory conditions. In the illusory condition, however, we expected a subsequent recovery of beta dominance, indicating a resolution of the mismatch due to phonetic alignment. Finally, we trained a Machine Learning (ML) algorithm to investigate whether it was possible to classify two language classes (English and Italian) from neural activity.

## 2 Materials and Methods

### 2.1 Participants

A total of 21 volunteers healthy native Italian-speaking participants (mean age = 21.96 years, SD = 1.43) were recruited for this study. The number of subjects was chosen as similar studies (Sohoglu & Davis, 2016). All participants were university students accustomed to using English on a daily basis. The sample included nine female participants. All participants were native Italian. This population was specifically chosen to investigate whether non-native listeners could experience cross-linguistic perceptual illusions driven by phonological similarity between languages. The illusion was, therefore, expected to arise primarily from the interplay between phonological similarity and top–down expectations shaped by the text prime, rather than from explicit semantic knowledge of the English lyrics. Participants were recruited and recorded at the University of Ferrara, where the experimental sessions took place in a sound-attenuated room to ensure optimal recording conditions. Participants were informed about the experimental procedure and gave their written consent according to the 1964 Helsinki Declaration, as revised in 2013. None of the participants reported neurological, psychiatric, or other contraindications to the experimental procedure. The study and experimental procedures were approved by the local ethics committee (Comitato Etico di Area Vasta Emilia Centro, approval number: *EM255-2020-UniFe/170592-EM Estensione*).

### 2.2 EEG acquisition and task procedure

EEG data were recorded using a 64-channel actiCHamp Plus EEG system (Brain Products GmbH, Gilching, Germany). Electrodes were placed according to the international 10–20 system with reference electrode at left mastoid and ground at AFz position. The impedance was kept below 10 kΩ, and the sampling rate was set to 1000 Hz. Participants were seated approximately 60 cm from a computer screen in a dimly lit room to minimize visual distractions.

The study protocol consisted of three main parts: two audiobook-listening tasks and the main experimental task. During the audiobook-listening tasks, participants were asked to listen to an 11 min English or Italian audiobook fragment—one before and one after the experimental task. The order of English and Italian audiobook presentations was counterbalanced across the sample. The data recorded during the audiobook phases were then used to train classifier.

**Fig. 1.**
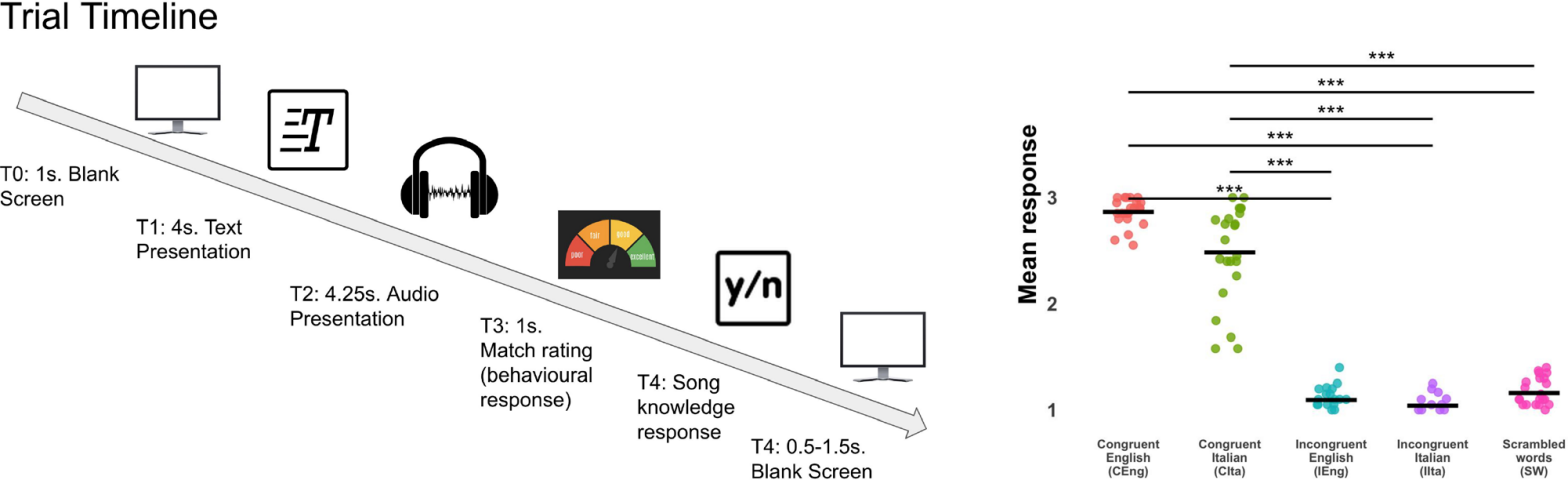
Experimental paradigm and behavioral responses. The left panel illustrates the timeline of each trial. It consisted of five sequential phases. At T0, a blank screen was presented for 1 s to center attention. At T1, a linguistic prior (a short text sentence) was displayed for 4 s. This was followed at T2 by the auditory target stimulus—an English song fragment (average length 4.25 s). At T3, participants rated the perceived match between the text and the audio on a 3-point scale (1 = poor, 2 = fair, 3 = excellent). At T4, they indicated whether they recognized the song (yes/no). A variable inter-trial interval (0.5–1.5 s) followed before the onset of the next trial. This design allowed us to test whether textual cues (priors) influence auditory perception under varying conditions of linguistic congruence and plausibility. The right panel shows behavioral results. A Friedman test revealed a significant effect of condition on response values. Pairwise Wilcoxon signed-rank tests with Bonferroni correction were used for post hoc comparisons, and significance levels are indicated by asterisks: (***p < 0.001).

After hearing one of the two audiobooks, the main experimental task began. The structure of the experimental trials is illustrated in Figure 1. Each trial started with the presentation of a fixation point to direct participants’ attention. Subsequently, a text prior was displayed for 4 s, which participants were instructed to read attentively. Following a brief pause (between 0.5 and 1.5 s), participants listened to a fragment of an English song (mean duration = 4.21 ± 3 s). Note that the text prior was not present during the presentation of the audio fragment. After hearing the audio fragment, participants rated the match between the text they had read and the audio they had heard on a 3-point Likert scale (1: “not at all” to 3: “very much”). Participants were instructed to focus only on phonological similarity and not on semantic similarity. After that, we asked participants to respond as quickly as possible. Finally, they indicated whether they recognized the song (“Yes” or “No”).

The experimental task consisted of three sequential phases. Participants first completed a practice block of 10 trials to become familiar with the procedure. This was followed by 2 main experimental blocks of 50 trials each, totaling 100 trials. To reduce fatigue and maintain engagement, a short self-paced break was provided between the 2 blocks. Within each block, trials were presented in a fully randomized order, and this order was further randomized across participants to minimize order and learning effects.

Each trial involved two key components: a textual prior (T1), displayed on screen, followed by an auditory stimulus (T2), which participants listened to through headphones. The auditory stimulus was a short (1–4 s) excerpt extracted from a unique English-language pop song, with no repetitions across the experiment. These excerpts were carefully selected so that, when preceded by a linguistically suggestive prior, they could plausibly be perceived as phonetically similar to Italian phrases by native Italian listeners. For example, a fragment containing the English phrase “you believe in love” could be misheard as “io vivo in là” when primed by a phonetically similar Italian text. All stimuli were piloted and screened to ensure their potential to generate cross-linguistic perceptual illusions. Given the natural variability inherent in real-world music, the auditory fragments differed in duration, time, and phonological complexity.

The entire task was implemented using PsychoPy (Peirce, 2007), enabling precise control over stimulus timing and presentation across trials and participants.

The 100 trials were equally divided among 5 experimental conditions, each designed to manipulate the relationship between the prior text and the auditory stimulus in a distinct way. In the Congruent English (CEng) condition, the prior text consisted of the exact lyrics that would follow in the English audio fragment, providing a direct match. In the Congruent Italian/Illusory (CIta) condition, the prior text was an Italian sentence that did not match the actual lyrics semantically, but was phonetically similar to them, potentially inducing the illusion that the upcoming English lyrics were in Italian. The illusion effect was created using some particular stimuli called in Italian “*canzoni travisate*” or “*misheard songs,*” that induce phonological similarity. The songs were selected from a large number of files available on the internet, particularly on YouTube. Songs and stimuli were selected that had the greatest possible similarity at the onset of the stimulus because, especially with regard to EEG analysis of ERPs, we focused on the time points immediately after onset. We chose to set the onset at the beginning of the auditory stimulus because we are interested on generally phonological similarity effect not on semantic similarity. We, therefore, focused our analyses on the early portion of the lyrics, which represents the most conservative choice when using naturalistic stimuli, as variability in word length and inter-word timing across songs makes reliable alignment to later words increasingly difficult and potentially introduces unwanted temporal jitter. In the Incongruent English (IEng) condition, the prior text was a grammatically correct English phrase taken from a different song, creating a semantic mismatch within the same language. In the Incongruent Italian (IIta) condition, the prior was an Italian sentence unrelated both semantically and phonetically to the upcoming English audio, serving as a baseline for mismatch in the participants’ native language. Finally, in the Scrambled Words (SW) condition, the prior consisted of pseudo-random letter strings, devoid of any semantic or phonological content, thereby eliminating any meaningful contextual influence.

### 2.3 EEG data pre-processing and analysis

The EEG data acquired during the audiobook and main experimental tasks were pre-processed using the same pipeline to ensure consistency across all neural signals.

For pre-processing firstly, Raw EEG signals were downsampled to 250 Hz. Channels was re-referenced to the average of the two mastoid electrodes (M1, M2). The signal was filtered using a finite impulse response (FIR) filter with a passband from 0 to 125 Hz. To identify and remove ocular and muscular artifacts, independent component analysis (ICA) was performed using the EEGLAB runica algorithm (Delorme & Makeig, 2004). Independent component analysis (ICA) was performed using the Infomax algorithm as implemented in EEGLAB. Artifactual components were identified with the ICLabel classifier (Pion-Tonachini et al., 2019), which provides probabilistic estimates for categories such as ocular, muscular, cardiac, line noise, channel noise, and brain-related activity. To adopt a conservative approach, we removed only those components for which ICLabel assigned an artifact probability between 0.90 and 1.00 in any non-brain category. This ensured that only components with a high likelihood of reflecting non-neural activity were rejected, while preserving ambiguous or mixed components that might contain residual neural information. An average of 9.5 ± 5.6 components were removed. In addition, a visual inspection was conducted to reject epochs containing residual artifacts. The cleaned EEG data were first segmented into epochs time locked to the onset of the auditory stimulus and baseline corrected using the mean activity in the pre-stimulus interval (-200 to 0 ms). To isolate neural responses related to linguistic processing and minimize confounding effects associated with the physical onset of the music, epochs were then realigned to the onset of the first sung word of each song, thereby reducing early sensory responses driven by music onset rather than by speech-related content.

For the analysis of ERP components, continuous EEG data were epoched from -200 ms to 350 ms relative to the onset of the auditory stimulus; specifically, stimulus onset was defined as the beginning of the first word of the song fragment for each trial. Baseline correction was applied using the -200 to 0 ms pre-stimulus interval. We selected a region of interest (ROI) comprising fronto-central-parietal electrodes (Liu et al., 2012; Luck, 2014; Näätänen & Alho, 1995; Polich, 2007). The fronto-central-parietal ROI was selected based on the well-established scalp distribution of the N1 and P2 components, which typically show maximal amplitudes over fronto-central areas with extensions toward centro-parietal sites in auditory and speech processing studies (Crowley & Colrain, 2004; Näätänen & Picton, 1987). The ROI included electrodes Fz, FCz, Cz, CPz, and Pz, and waveforms were averaged across these sites to obtain a robust measure of early auditory responses. The same ROI was used for oscillatory analyses to ensure direct comparability with ERP results, as theta and beta modulations during speech perception often arise over similar fronto-central regions (Luo & Poeppel, 2007; Peelle et al., 2013).

We focused on two canonical ERP components associated with auditory prediction: the N1, defined within the 100–150 ms time window, and the P2, defined within the 150–250 ms interval.

Statistical differences between conditions were evaluated using independent-samples t-tests at each time point across participants. To correct for multiple comparisons across time, we applied a non-parametric cluster-based permutation test (1000 iterations) to identify significant temporal clusters of condition differences. In complementary analyses, condition-wise ERP amplitudes were also averaged within pre-defined time windows (N1: 100–150 ms; P2: 150–250 ms) and subjected to repeated-measures ANOVA.

Additionally, to assess oscillatory dynamics associated with predictive processing, time–frequency decomposition was performed using Morlet wavelet convolution (7 cycles) from 2 to 80 Hz in 1 Hz steps. Power values were computed for each trial and condition, and normalized by converting to decibel (dB) change relative to the –200 to 0 ms pre-stimulus baseline. This analysis was performed on the same fronto-centro-parietal ROI used in the ERP analysis, with power values subsequently averaged across these electrodes.

We focused on the beta (13–30 Hz) and gamma (30–50 Hz) frequency bands, based on their established roles in top–down predictions and bottom–up prediction errors, respectively (Arnal et al., 2011; Bastos et al., 2012). In contrast to our ERP analysis, the time window of interest for this analysis was set to 0–800 ms post-stimulus onset. This difference was because our ERP analysis focused on early phonological processing, while our time–frequency analysis was intended to investigate more semantic processing. For analyses involving the beta–gamma ratio, our aim was to characterize how the relative contribution of beta- and gamma-band activity varied across experimental conditions. Rather than interpreting the ratio in terms of deviations from a fixed reference value (e.g., 1), we used it as a functional index of the balance between beta-related top–down predictive activity and gamma-related bottom–up prediction error signaling. In this framework, higher values of the beta–gamma ratio reflect a predominance of predictive signals, whereas lower values indicate increased prediction error activity. Accordingly, the ratio provided a compact and interpretable measure of how the interplay between these two frequency bands changed as a function of the task context.

Statistical analysis of power differences across conditions was conducted using nonparametric cluster-based permutation testing (1000 permutations). Time–frequency representations were first computed at the single-subject level, and power values were then entered into paired comparisons across conditions. To control for multiple comparisons across time and frequency, clusters of adjacent time–frequency points exceeding a pre-defined threshold (*p* < 0.05) were identified, and cluster-level statistics were evaluated against a null distribution obtained by randomly permuting condition labels across subjects. Significant effects were defined as clusters whose summed test statistics exceeded the 95th percentile of the permutation distribution. This approach provides robust control of family-wise error rates while preserving sensitivity to temporally and spectrally contiguous effects. All analyses were conducted in MATLAB.

### 2.4 Classifier analysis

In addition to the EEG analyses detailed above, we performed a classification analysis using a machine learning algorithm to achieve the best possible results. We built a classifier to distinguish between participants’ EEG signals associated with hearing English or Italian stimuli. The classifier was trained exclusively on EEG data collected while participants listened to the English and Italian audiobooks. The EEG signals were band-pass filtered between 0 and 40 Hz using a 4th-order Chebyshev filter and segmented into 2.5-s epochs. Data from all participants were pooled together. A processing pipeline based on Common Spatial Patterns (CSP) followed by Mutual Information (MI) was then applied to extract the most informative spatial features (Frosolone et al., 2024). CSP, commonly used in EEG signal analysis for spatial filtering, generates a lower-dimensional space that enhances the separability of labeled samples from distinct classes. MI, an information-theoretic measure, quantifies the statistical dependency between two random variables. In this context, MI was employed to select the spatial features carrying the most discriminative information for classification. The features extracted through the CSP-MI pipeline were used to train a Quadratic Discriminant Analysis (QDA) classifier to distinguish between English and Italian speech. QDA was chosen due to its ability to model class-specific covariance structures, thereby better capturing the variability in EEG patterns associated with distinct auditory stimuli (Frosolone et al., 2024). Using a Leave-One-Subject-Out classification strategy, with a repeated k-fold cross-validation with k = 10 applied within each training set for model optimization, an accuracy of 77.9 ± 2.8% was obtained in distinguishing between English and Italian audiobook-related EEG signals. To clarify the classification procedure, mutual information (MI) was computed between each CSP-derived spatial feature and the class labels (English vs. Italian). MI, therefore, quantified how strongly the distribution of each spatial projection covaried with the stimulus language, allowing us to retain only those components carrying the highest discriminative value. The classifier did not incorporate fine-grained temporal dynamics of the individual audio stimuli; instead, each 2.5-s epoch was treated as a multichannel spatial pattern, consistent with standard CSP-based pipelines in auditory EEG decoding. This approach enabled the model to learn spatially distributed patterns of oscillatory activity rather than stimulus-specific temporal trajectories. Although CSP filters are spatial by construction, they exploit differences in spatial covariance structure that inherently reflect stable spatio-temporal dynamics across trials, making the resulting features sensitive to consistent topographical patterns distinguishing English from Italian speech. Such spatially informed components are among the most robust and interpretable EEG features, particularly in cross-subject classification settings (Blankertz et al., 2008). The classifier, therefore, relied on spatial–spectral patterns characteristic of the two languages, rather than on stimulus-locked acoustic differences, supporting its application to the song fragments. Consistent with this view, prior work by Maess et al. (2012) has shown substantial overlap in the cortical networks involved in linguistic structure and melodic contour processing, particularly within superior temporal and frontotemporal regions. Classification performance was computed at the trial level across all participants pooled together, rather than at the single-subject level. Each trial was treated as an independent observation and assigned a binary label (Italian vs. English). For each experimental condition, accuracy was calculated as the proportion of correctly classified trials over the total number of valid trials aggregated across participants, after artifact rejection. Consequently, the reported classification scores reflect global trial-wise decoding performance rather than inter-individual variability in accuracy. In practical terms, each participant contributed approximately the same number of trials per condition (about 20), but performance estimates were derived from the pooled dataset (approximately 20 × *N*_participants_ trials per condition, minus rejected trials), yielding a single accuracy value for each condition. This analytical choice was motivated by the goal of assessing whether stable, language-related spatial–spectral patterns generalize across individuals, rather than by the aim of characterizing subject-level differences in decoding performance.

The trained classifier was then applied to EEG data recorded during the five experimental conditions. For each condition and for each of the 20 trials, EEG signals were averaged across participants on a channel-by-channel and time-point basis, resulting in 20 group-averaged trials per condition. This processing step aimed to minimize inter-subject variability. The averaged trials were then projected onto the manifold defined by the CSP-MI features extracted from the audiobook data. Subsequently, the trained QDA classifier was used to assign a class label (English or Italian) to each trial. For each condition, the percentage of predictions per class (i.e., the percentage of trials classified as English or Italian) was computed.

## 3 Results

### 3.1 False but phonologically plausible linguistic priors induce auditory illusions

We assessed how linguistic priors influenced perceived congruence between text and auditory stimuli. A non-parametric Friedman test was conducted to assess differences in ordinal responses across the five experimental conditions: CEng, CIta, IEng, IIta, and SW. The test revealed a significant main effect of condition, χ^2^(4) = 78.7, *p* < 0.001, indicating that participants’ responses varied significantly depending on stimulus type.

Post hoc pairwise Wilcoxon signed-rank tests with Bonferroni correction showed that responses in the CEng condition significantly differed from those in CIta (*p*_adj_ = 0.12), IEng (*p*_adj_ < 0.001), IIta (*p*_adj_ < 0.001), and SW conditions (*p*_adj_ < 0.001). Moreover, CIta responses were also significantly different from IEng (*p*_adj_ < 0.001), IIta (*p*_adj_ < 0.001), and SW (*p*_adj_ < 0.001). A significant difference was also found between IIta and SW (*p*_adj_ = 0.003). In contrast, no significant differences were observed between IEng and IIta (*p*_adj_ = 0.638), or between IEng and SW (*p*_adj_ = 0.088). To control whether there could be a time-to-task effect in subject response, caused by the learning of the presentation of all English songs, we used a linear and a ordinal logistic model. Both the linear and ordinal logistic models revealed no significant effect of trial order on response level in the CIta condition (linear model: *p* = 0.76; ordinal model: *p* = 0.66). Participants’ responses remained stable across the task, indicating no evidence of a time-on-task effect. Finally we check the effect of song knowledge: We found no statistically significant effect of participants’ song familiarity on their ratings in the CIta condition.

### 3.2 A classification analysis supports the auditory illusion

We next investigated whether it would be possible to infer whether participants were experiencing an auditory illusion from their brain activity. To address this, we trained a classifier to distinguish between neural activity associated with hearing English or Italian stimuli, using the EEG signals recorded while participants listened to audiobooks in the two languages—and tested the same classifier on the EEG signals recorded during the five experimental conditions (see Section 2.4 for details).

We classified the EEG data from each of the five experimental conditions separately. We used the classification accuracy per class (i.e., the percentage of times the classifier assigned the EEG data recorded during the five experimental conditions to either the English or Italian class) to assess whether participants’ brain responses resembled those typically associated with hearing English or Italian speech.

The results of this analysis are presented in Table 1. Notably, CIta trials were more frequently classified as Italian, providing support for the auditory illusion. The other trials were more frequently classified as English. We repeated the analysis after excluding the small subset of CIta trials in which participants did not report the illusory perception (approximately 40 trials in total), classification performance remained biased toward Italian responses (about 55% vs. 45%), but overall accuracy decreased due to the substantially reduced number of trials available for EEG-based decoding.

**Table 1.** Classification results.

Percentage of times the assigned class is English or Italian [%]		
Condition	English	Italian
Congruent English	**65**	35
Congruent Italian	35	**65**
Incongruent English	**55**	45
Incongruent Italian	**55**	45
Scrambled words	**70**	30

Classification accuracy for each condition (i.e., percentage of times the classifier assigns the EEG data recorded during the five experimental conditions to either the English or the Italian class). Bold values indicate the predominant classification outcome for each experimental condition.

### 3.3 Phonological plausibility linguistic priors modulate surprise and prediction error signals in auditory ERP components

#### 3.3.1 N1 responses reflect prediction error signals

In addition to the P2, we observed an early fronto-central negativity across several contrasts, peaking around 100 ms, consistent with the N1. This component has been linked to automatic detection of violations in expected auditory input and is widely interpreted as a neural correlate of prediction error (Taylor et al., 2024).

**Fig. 2.**
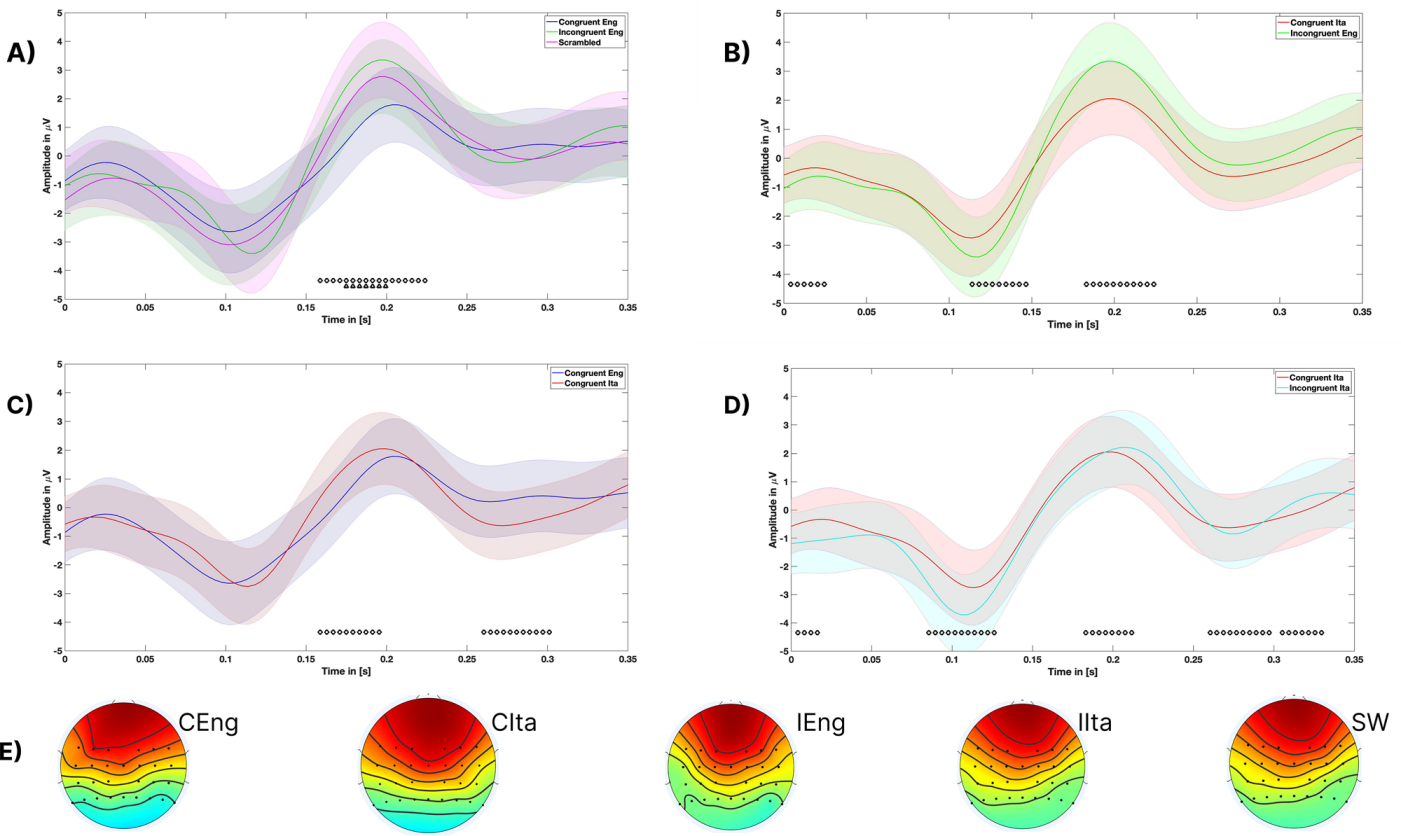
Topographic maps of ERP responses. Panels (A–D) show the differences in the amplitudes over time of the ERPs between 0 and 800 ms after the onset of the auditory stimulus. Panel A shows the differences between the CEng IEng and SW conditions: Colored dots below the x-axis indicate time windows of significant pairwise differences: black = CEng vs IEng, red = CEng vs. SW; Panel (B) shows the differences between the CIta and IEng conditions; Panel (C) shows the differences between the CEng and CIta conditions; Panel (D) shows the differences between the CIta and IIta conditions; Panel (E) shows the average topographic activation of the five conditions, calculated between 150 ms and 250 ms after onset. The absence of a baseline in the plots is due to the shift of the onset to the beginning of the “speech.” Therefore, before the onset, pieces of music may be present for some stimuli, and thus there is no true baseline.

In Figure 2. IEng and SW show numerically larger, though non-significant, N1 amplitudes than CEng. Figure 2B shows a significantly greater N1 for IEng than for CIta, despite both involving incorrect priors. This pattern suggests that phonological plausibility in CIta may attenuate early auditory responses, potentially by engaging partial matches to expected acoustic patterns.

Figure 2C, comparing CEng and CIta, reveals highly similar early ERP profiles, with no significant N1 differences. This further supports the idea that CIta engages prediction mechanisms similarly to CEng, despite its illusory nature.

In Figure 2D, the CIta condition elicits a greater early negativity than IIta, suggesting that the phonologically implausible structure of the IIta condition fails to engage predictive mechanisms as robustly as CIta. The difference highlights that the violations of expectations revealed by our study do not merely reflect an incorrect language.

Together, these N1 results suggest that the brain generates prediction and prediction error signals early in processing, possibly reflecting simple (e.g., acoustic) features of the musical stimulus, and that these signals are modulated by both the presence and the plausibility of linguistic priors.

#### 3.3.2 P2 responses reflect graded sensitivity to auditory surprise

Figure 2 presents grand average ERP waveforms and topographical maps for the critical contrasts between experimental conditions, time locked to the onset of the song. Figure 2A–D shows comparisons across conditions, while Figure 2E displays topographic distributions averaged from 150 to 250 ms.

All ERP analyses were conducted on grand-averaged waveforms extracted from a fronto-centro-parietal region of interest (ROI). The signals were averaged across all participants and electrodes within this ROI. The colored horizontal markers below the waveforms indicate time points at which statistically significant differences in signal amplitude between conditions emerged, based on independent-samples t-tests (*p* < 0.05).

In Figure 2A, a peak (around 200 ms) response is most pronounced in the IEng and SW conditions compared with the CEng condition. This suggests that both lexical violations and lack of structure increase the prediction error response, in line with the P2 component indexing attentional orienting to unexpected stimuli (Kanske et al., 2011).

Figure 2B shows a clear distinction between IEng and the Illusory CIta, with IEng eliciting a higher peak (around 200 ms) amplitude. This indicates that phonologically plausible priors—as in the CIta condition—can reduce neural surprise despite being incorrect, likely due to partial matching with predicted input.

Figure 2C compares CEng and CIta. Although both conditions are associated with congruent expectations, CIta elicits a slightly larger peak (around 200 ms), reflecting the qualitative difference between true and illusory congruence. This difference suggests that even when phonological expectations align, false priors may still generate mild prediction error signals.

Figure 2D compares CIta and IIta. Here, the IIta condition elicits a significantly stronger peak (around 200 ms) than CIta, reinforcing that the effects observed in CIta cannot be attributed to the Italian language alone. Rather, the attenuation of prediction error in CIta is likely due to the phonological plausibility of the illusory match. An additional comparison reveals a smaller peak (around 200 ms) in the IIta than the IEng condition, suggesting that linguistic priors in a different language, even when incongruent, generate weaker expectations and consequently reduced prediction errors. Note that Figure 2C and 2D also shows significance differences between waveforms around 300 ms, which were not hypothesized and remain to be investigated in future studies.

**Fig. 3.**
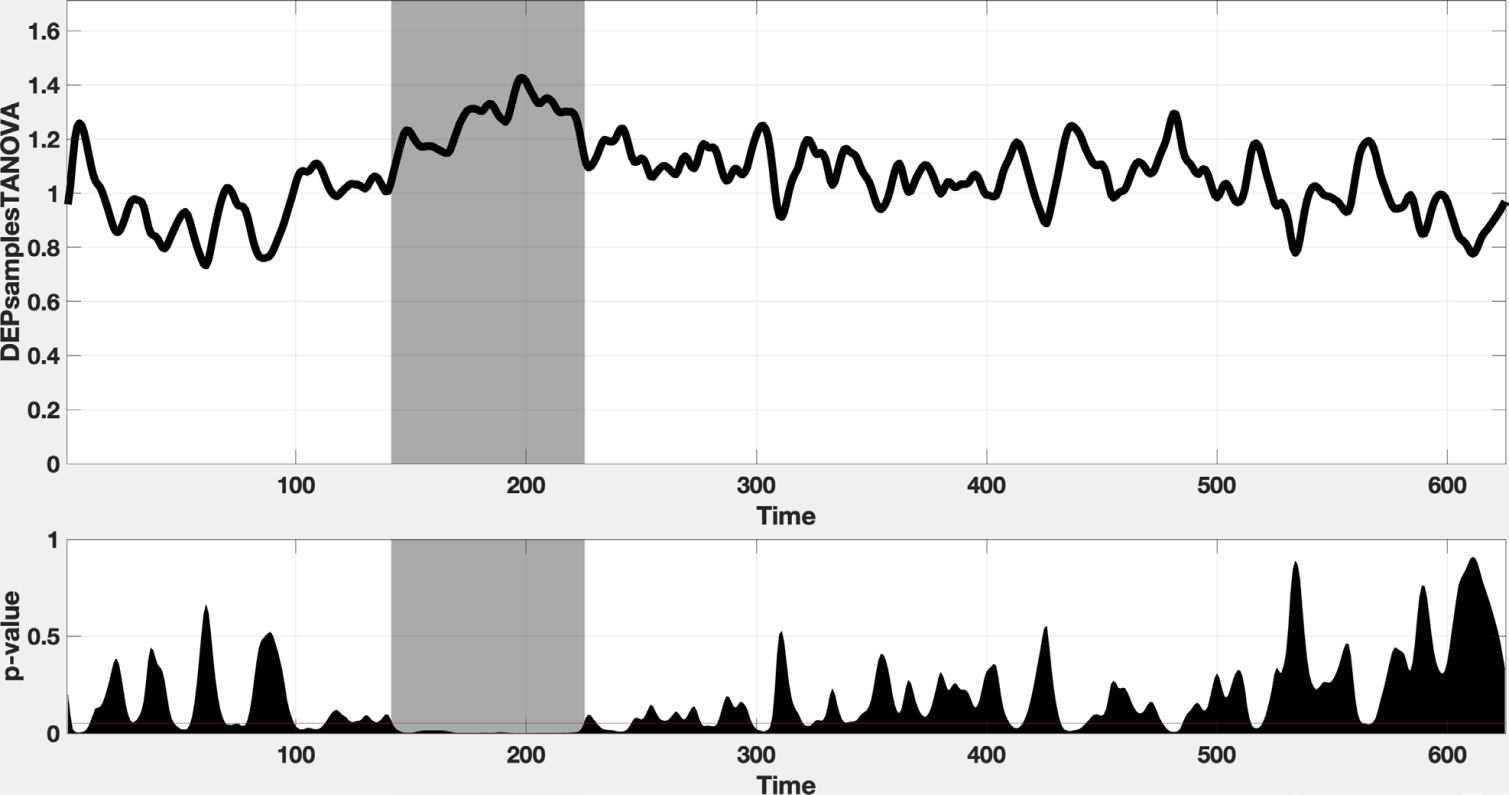
Time-resolved topographic ANOVA (TANOVA). The upper panel shows the Global Dissimilarity (DISS) between conditions across the 0–600 ms window; the shaded region marks the predefined time interval of interest. The lower panel displays permutation-based *p*-values (*α* = 0.05, red line). A clear cluster of significant topographic differences emerges within the highlighted window, indicating distinct scalp configurations between conditions during this period. A significant cluster spanning approximately 150–225 ms post-stimulus onset was evident (highlighted window), indicating that the spatial distribution of EEG responses differed between conditions.

Finally, Figure 2E shows topographic maps of mean ERP amplitudes. In particular, as shown in Figure 3, we run a TANOVA test (Koenig & Melie-Garcia, 2011) and we found a significance differences around 150–250 ms time window across the five experimental conditions and we chose that time interval to plot the maps. Most conditions exhibit a fronto-central positivity consistent with the P2 component, but are modulated by condition. CEng and CIta show the most focal fronto-central activation, suggesting efficient engagement of attention mechanisms. In contrast, IEng exhibits a more diffuse topography, IIta is characterized by attenuated fronto-central positivity accompanied by a posterior negativity, and SW shows the weakest activation overall. These scalp distributions are consistent with the notion that both the linguistic content and phonological plausibility of the prior modulate the amplitude and spatial extent of the P2 response. To see the ERP comparison of all 5 conditions, see Figure S1 in Supplementary materials.

### 3.4 Auditory illusions show a transition from gamma-dominated to beta-dominated activity

Our next analysis focused on oscillatory activity in the high-beta and low-gamma frequency bands, as these have been strongly implicated in predictive coding and predictive routing. Specifically, beta oscillations (~15–30 Hz) have been associated with the maintenance of top–down predictions and the stability of perceptual representations, whereas gamma oscillations (~30–80 Hz) have been linked to the encoding of prediction errors, signaling the need for an update in internal models when incoming sensory information deviates from expectations (Arnal et al., 2011; Bastos et al., 2020, 2012; Friston et al., 2015).

**Fig. 4.**
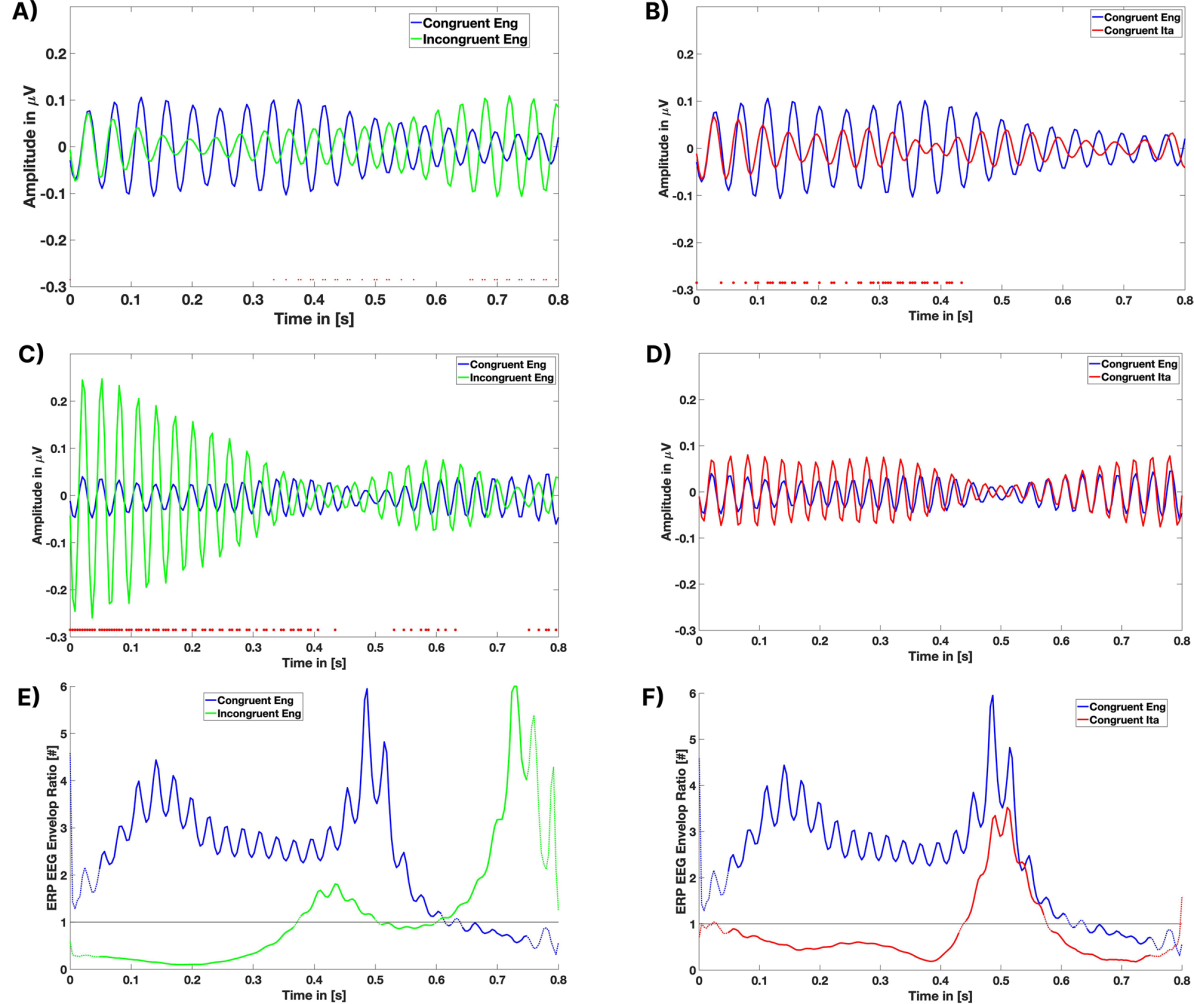
Time–frequency analysis of EEG responses and EEG Envelop Ratio. Panels (A–D) illustrate time–domain analyses in different frequency bands: beta high (A, B) and gamma low (C, D), comparing “Congruent English” (blue) and “Incongruent English” (green) and “Congruent Italian” (red) and EEG signals. Each panel highlights how amplitude evolves over time in the respective band, revealing distinct temporal patterns between the two conditions. Panels (E–F) focus on the dominance of the beta/gamma ratio, showing how this metric varies over the same time window for both conditions: when the ratio exceeded 1, beta oscillations were dominant; when below 1, gamma oscillations prevailed. The comparison provides insight into how congruence modulates neural oscillatory dynamics across frequency ranges.

Figure 4A and C shows the analysis of high-beta and low-gamma bands activity when comparing the congruent (CEng) and incongruent English (IEng) conditions. As mentioned before, a point-by-point *t*-test was performed to assess amplitude variations over time. Red markers indicate time points where significant differences were observed (*p* < 0.05). The analysis shows an anti-correlated trend between the two bands, with the CEng condition eliciting stronger beta oscillations in interval 0.4–0.6 s and the CIta condition eliciting stronger gamma oscillations across almost all the intervals from 0 to 0.6 s. These findings are compatible with the idea that the congruent condition elicits stronger top–down prediction (in the high-beta band) and conversely the incongruent condition elicits violations of expectation (in the low-gamma band).

A different pattern emerges when comparing the congruent (CEng) and illusory (CIta) conditions (Fig. 4B and D). The CEng condition elicits stronger beta oscillations than the CIta condition, but there is no difference in the gamma activity between these two conditions. The finding that gamma activity is significantly enhanced in the incongruent but not in the illusory conditions highlights a significant difference between the two processes, with the former potentially increasing the precision of sensory stimuli to overcome an unreliable prior and the latter concerned with the assimilation of prior and stimuli to form an illusory percept.

To further explore these differences, we examined the ratio between beta and gamma power across conditions to determine which frequency range was more dominant over time (Fig. 4E-F; see also Figure S2 in Supplementary materials for additional plots). For each subject, the EEG signals were filtered in the frequency bands of interest. Subsequently, only the channels corresponding to the frontal-central parietal ROI were extracted. The data were averaged per condition across all trials and then across the selected channels, with each frequency band processed independently. Next, the Hilbert transform (Boashash, 1992) was computed for the resulting ERP in each band, and its magnitude was extracted. The ratio between the beta and gamma amplitude envelopes was then calculated. Finally, we plotted continuous lines to indicate all time points where the beta/gamma ratio deviated from unity for an interval longer than half of the selected time window (i.e., 100 ms). When the ratio exceeded 1, beta oscillations were dominant; when below 1, gamma oscillations prevailed.

Figure 4E shows the results of the comparison between the congruent (CEng) and incongruent (IEng) conditions. Early on, beta activity dominates in the CEng condition, reflecting the influence of the correct linguistic prior. In contrast, gamma activity initially dominates in the IEng condition, suggesting a heightened prediction error response that facilitates model updating. At a later stage, an inversion of dominance is observed in IEng, with beta activity overtaking gamma, potentially reflecting successful model updating and the reestablishment of predictive guidance in song perception.

Interestingly, in the CIta condition (Fig. 4F), there is an initial phase of gamma dominance (as in the IEng condition) followed by beta dominance, which occurred earlier than in the IEng condition. This pattern indicates that illusory congruence does not elicit the same neural response as true perceptual violations. Rather, they suggest that an initial phase of surprise is followed by an illusory “assimilation” and cross-linguistic integration of the prior (in Italian) and the song (in English). These findings also suggest that gamma oscillations dominate in surprising situations requiring model updating, while beta activity dominate in the presence of an expected patterns, even if illusory.

**Fig. 5.**
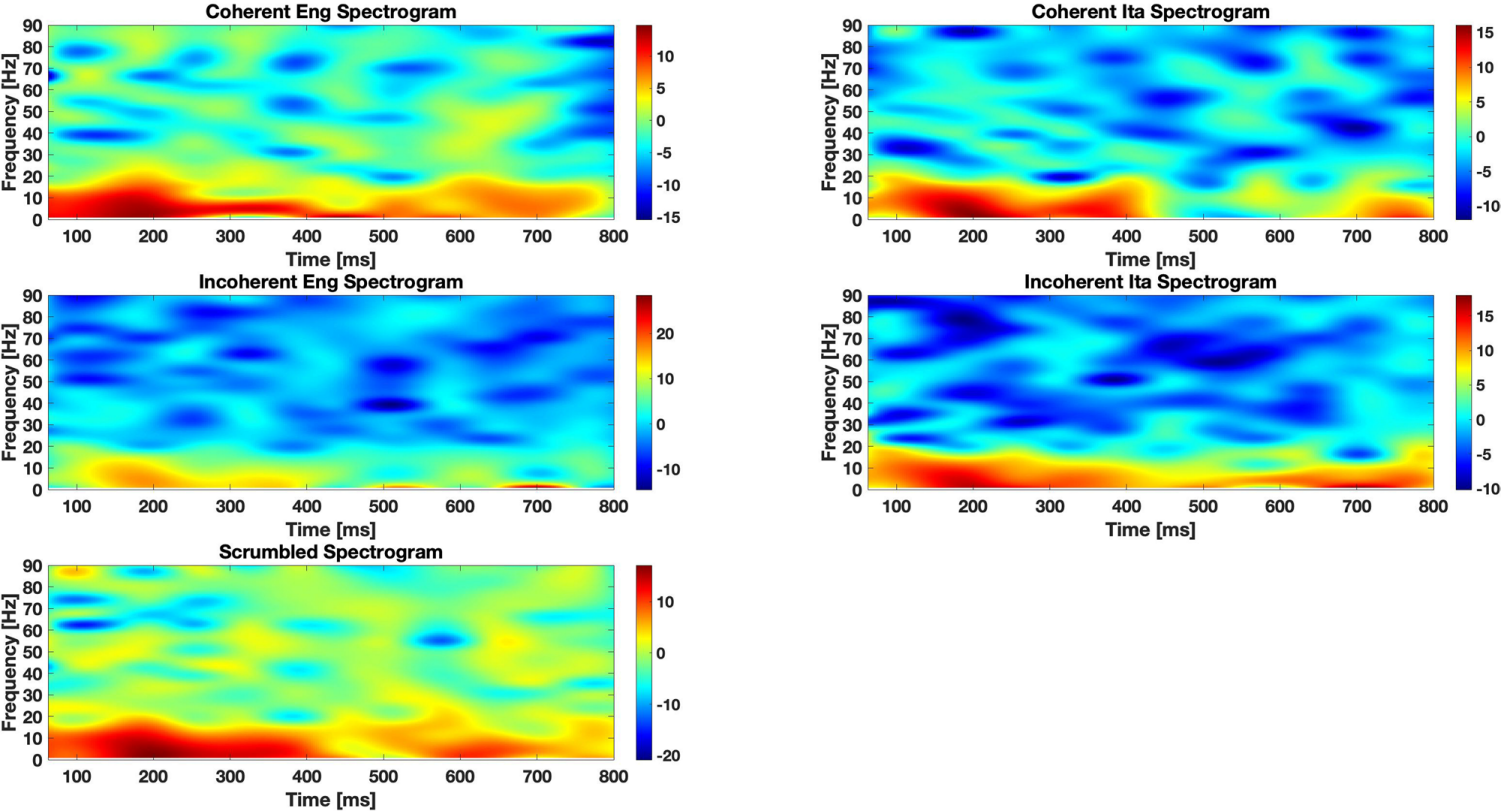
Time–frequency representations across conditions. Group-level spectrograms computed from stimulus-locked EEG activity (0–800 ms) for all experimental conditions. Each panel shows power changes (in dB) relative to baseline across frequencies from 1 to 90 Hz, derived using Morlet wavelet decomposition. Warmer colors indicate event-related power increases, whereas cooler colors reflect power decreases. Despite similar low-frequency structure across conditions, notable differences emerge in the beta–gamma range, where distinct temporal patterns of desynchronization and resynchronization can be observed. These patterns suggest condition-specific engagement of higher-order auditory and predictive processing mechanisms.

Finally, in line with the spectrotemporal patterns illustrated in Figure 5, the time–frequency analysis revealed condition-dependent modulations across low- and high-frequency ranges. While all conditions showed a comparable increase in low-frequency power shortly after stimulus onset, more pronounced divergences emerged at higher frequencies, particularly within the beta–gamma range (20–70 Hz). These modulations follow distinct temporal trajectories across conditions, suggesting differences in the recruitment of auditory prediction and integration mechanisms. Importantly, such spectrotemporal signatures are consistent with the hypothesis that listeners engage distinct neural strategies depending on the degree of congruence between the preceding text and the auditory input.

## 4 Discussion

Can English songs be perceived as Italian when accompanied by appropriate linguistic priors? How are the brain’s predictive mechanisms engaged during such auditory illusions?

This study examined how predictive coding mechanisms operate in auditory perception when linguistic priors create congruent, incongruent, or illusory expectations about upcoming auditory stimuli. By analyzing event-related potentials (ERPs) and time–frequency dynamics (specifically, high-beta and low-gamma frequency bands), we aimed to provide insights into how phonological congruence modulates neural responses in a naturalistic auditory task consisting of listening to songs with English lyrics. The variability in the structure and timing of the experimental trials was intentionally preserved, as it mirrors the natural diversity of real-world listening experiences. Rather than being treated as noise, this variability was considered an inherent feature of the task, enhancing its ecological validity and allowing a more realistic investigation of auditory perception in naturalistic conditions.

Our results show that when participants are given Italian linguistic priors that are phonologically congruent with an English song—presented before, but not during, the audio—they report an illusory perception of hearing Italian words. A classifier trained to distinguish electrophysiological signals associated with English versus Italian stimuli reported the congruent and incongruent conditions to be closer to English stimuli, but the illusory condition to be closer to Italian stimuli. These findings extend previous studies on predictive coding during auditory perception, demonstrating that false but phonologically plausible linguistic priors can create cross-linguistic auditory illusions during naturalistic perception (Bonetti et al., 2022, 2024; Clark, 2013; Koelsch et al., 2019; Lupyan & Clark, 2015; Parr et al., 2022; Popescu et al., 2024).

To investigate in more detail the electrophysiological responses elicited during the perception of congruent, incongruent, and illusory lyrics, we focused on two early ERP components, N1 and P2, associated with surprise or prediction error during low-level auditory and phonological processing (Crowley & Colrain, 2004; Tast et al., 2024). As expected, the congruent condition elicited the smallest modulation of N1 and P2 components, suggesting small prediction error due to a strong match between prior expectations and auditory stimuli. However, sources of uncertainty (such as varying inter-trial intervals and random trial order) could explain residual prediction errors in these “baseline” conditions as CEng and SW. Rather, incongruent conditions were associated with greater modulation of N1 and P2 components, possibly reflecting greater prediction error. Crucially, the illusory condition was associated with a dampened modulation of N1 and P2 components compared with the incongruent condition, suggesting a partial matching with predicted auditory stimuli. Finally, the condition in which participants received uninformative (scrambled) linguistic priors showed relatively attenuated electrophysiological responses, possibly reflecting the fact that the uninformative context minimizes the formation of strong predictions and thus reduces the magnitude of the prediction error. Finally, a caveat for our interpretation is that we did not observe significant N1 differences between the CEng, IEng, and SW conditions, as would be expected if the N1 straightforwardly indexed prediction-error magnitude across all types of expectancy violations; the reasons for this null effect remain to be investigated and may reflect limited sensitivity of early auditory responses to certain forms of violation or an overlap between sensory-evoked and predictive processes at early latencies.

We next examined the relative dominance of beta and gamma rhythms as an index of prediction-based versus sensory-based processing. As hypothesized, the congruent condition was associated with the greatest predictive engagement, as indexed by a dominance of beta over gamma rhythms. These findings are consistent with previous work showing that beta-band activity is involved in transmitting top–down prediction signals, especially in speech and language processing (Arnal & Giraud, 2012; Spitzer & Haegens, 2017). In the low-gamma band, true incongruence elicited stronger oscillatory responses than the congruent condition, in keeping with previous interpretations in predictive coding and predictive routing that gamma-band activity reflects bottom–up signaling of unexpected sensory input and model updating (Bastos et al., 2020, 2012; Zhang et al., 2019). Similar effects have been reported in music cognition, where violations of melodic expectations increase gamma power, indicating neural computation of prediction errors (Koelsch et al., 2019; Mai & Wang, 2023).

Importantly, the illusory condition did not show the same enhanced gamma-band activity characteristic of the incongruent condition, despite the difference in the lexical content of the text prior and the song lyric. Furthermore, in the illusory condition, there was a relatively early recovery of predictive engagement (as indexed by beta dominance) during the trial. This result suggests that the illusory percept does not elicit enhanced sensory processing (possibly mediated by greater gamma activity) for model updating, but with an assimilation of the sensory signals into the illusory percept. Yet, the beta dominance observed in the illusory condition is weaker than that in the truly congruent condition. This aligns with findings that internally generated signals are generally weaker than veridical sensory input—suggesting that signal strength may help the brain distinguish between reality and imagination (Dijkstra & Fleming, 2023).

These results align with predictive coding and predictive routing frameworks, in which beta oscillations help maintain internal models, while gamma signals incoming errors (Bastos et al., 2020, 2012; Friston, 2005; Parr et al., 2022). The key novelty of this study is showing the crucial difference between the brain processes mediating incongruent conditions—dominated by large surprise and prediction error signals, as evident by P2 and gamma activity—and illusory conditions—showing dampened neural surprise despite the false prior. Our findings suggest that the illusory process might be mediated by the assimilation of the sensory stimulus to the false prior rather than a revision of the false prior. Typically, ERP markers of prediction and prediction error include the N2 and P3a components (Bekinschtein et al., 2009; Pinheiro et al., 2019). However, in our study, the most pronounced effects were observed earlier, around 150–250 ms (P2 time window). This temporal shift may be due to the highly naturalistic nature of our paradigm, which differs substantially from classical oddball tasks that are designed to optimize signal-to-noise ratios for detecting prediction errors. In contrast, our stimuli were all novel and acoustically rich, with each trial presenting a unique combination of text and song fragment. Furthermore, the musical component itself may modulate the morphology and timing of ERP waveforms, possibly enhancing early perceptual and attentional responses and reducing the distinctiveness of later components. While our study revealed attenuated P2 and gamma responses during illusory trials, suggesting reduced prediction error when linguistic priors dominate perception, recent ERP evidence points to a complementary mechanism at later processing stages. Silcox and Payne (2025) showed that during false hearing, the N400 response to phonological lure words was indistinguishable from that elicited by congruent words, indicating that listeners processed the lure as though it were the predicted word. Unlike our early markers of perceptual assimilation, this later facilitation effect suggests that false hearing may reflect a distinct, post-perceptual phase of predictive integration. Taken together, these findings suggest that linguistic prediction influences multiple stages of auditory processing—from early perceptual matching to later semantic access—and that the precise temporal locus of such effects may depend on the task demands and the strength of the prior. Moreover, our findings also resonate with recent studies demonstrating that false percepts in speech perception are driven by anticipatory activation of predicted lexical representations. For instance, Failes and Sommers (2022) used eye tracking to show that listeners’ gaze behavior reflects the pre-activation of predicted words even when those words are not actually presented, leading to confident but incorrect perceptions. This supports the view that auditory illusions, such as those observed in the present study, arise from top–down predictive mechanisms that can dominate early perceptual processing when sensory evidence is ambiguous.

While our findings offer new insights into predictive coding in speech and music, several questions remain. Future work should examine whether linguistic experience, musical training, or task difficulty affect how phonetic similarity shapes predictions (Corsini et al., 2024; Vuust et al., 2022). Furthermore, various studies indicate that language processing uses extensively predictive mechanisms (Altmann & Kamide, 1999; Caucheteux et al., 2023; Goldstein, Zada, et al., 2022; Kuperberg & Jaeger, 2016; Levy, 2008; Schrimpf et al., 2021). Exploring how these mechanisms operate across different linguistic and musical contexts could help clarify the boundaries and generalizability of predictive processing, as well as its role in shaping auditory illusions. Another direction involves clinical populations. Disorders such as tinnitus, schizophrenia, and dyslexia involve altered predictive coding, raising the question of whether sensitivity to phonetic illusions varies with these conditions (Barrett & Simmons, 2015; Donnarumma et al., 2023; Friston, 2023; Friston et al., 2016; Khalsa et al., 2018; Sedley et al., 2016).

Another open question concerns potential alternative interpretations of our ERP results. In our analysis, we focused on early components—specifically the N1 and P2—which have been previously linked to low-level auditory and phonological processing. However, within the same early time window (100–200 ms), another well-documented ERP component is the mismatch negativity (MMN), which has been robustly associated with prediction error (Garrido et al., 2009; Wacongne et al., 2011). This raises the question of whether the evoked potentials observed in our study might reflect MMN activity. However, MMN effects are typically elicited in paradigms involving repeated, identical standard stimuli interspersed with occasional deviants—a structure that contrasts with the naturalistic and variable stimuli used in our study. Another relevant early component is the P3a, which occurs slightly later than the P2. The P3a has been associated with attentional orienting to novel or unexpected events (Barceló, 2021; Barceló et al., 2002; Gómez et al., 2019; Turgeon et al., 2025), but it tends to be more pronounced in response to clearly deviant or salient stimuli. It remains an open question how our findings relate to prior work on these and potentially other ERP components that may reflect dynamic updates of predictions and prediction errors. The detailed discussion of this evidence offers solid heuristic grounds to reinterpret conventional ERP/EEG results from the novel predictive coding framework, and will also guide the pursuit of more fine-grained analyses in future studies.

In this study, all analyses were conducted on the full sample due to the relatively small size of the dataset. Future work should perform more fine-grained analyses, for example, by separating participants who truly experienced the illusion in the illusory condition from those who did not.

Furthermore, future work could explore other neural signatures of predictive processing in our paradigm. We emphasized beta and gamma bands due to their prominence in predictive coding theories. However, delta and theta rhythms may also play distinct roles, as shown by studies that linked the former to prediction errors and the latter to neural sharpening in speech perception tasks (Mai & Wang, 2023; Pastore et al., 2022). Moreover, we focused our analysis on early event-related potentials (ERPs), as these are closely linked to auditory predictive processing. This allowed us to examine the early stages in which linguistic priors are compared with auditory input in both congruent and incongruent conditions. It also enabled us to address our central question: What distinguishes congruent trials, incongruent trials, and illusory trials—where an initial violation of expectations is ultimately resolved, leading to an illusory auditory percept. Future work may extend this analysis to later phases of auditory processing in the EEG signal. However, this presents technical challenges, as our naturalistic paradigm requires presenting auditory stimuli only once, unlike typical EEG designs that rely on multiple identical trials. Despite these challenges, such analyses could offer deeper insight into the full time course of predictive processing during the perception of songs and lyrics that are congruent or incongruent with linguistic priors.

Finally, our paradigm using misheard lyrics might provide a compelling case for studying humor processing in auditory perception. Humor typically involves a two-stage cognitive process: initial detection of incongruity followed by reinterpretation (Canal et al., 2019). Future studies using our paradigm might address ERP markers of humor comprehension, such as the P600 component, reflecting reanalysis of incongruent input (Canal et al., 2019).

Summing up, this study provides evidence that false but phonologically congruent linguistic priors can create the auditory illusion to hear an English song in Italian. Furthermore, it shows that predictive coding mechanisms in auditory perception are differentially modulated by linguistic priors incongruent with the heard song versus those that generate auditory illusions. Neural surprise signals, associated with N1, P2, and gamma activity, are high with true incongruence—possibly reflecting the need to prioritize sensory information to update predictive models—and dampened in the case of auditory illusions—possibly reflecting a process in which sensory information is assimilated into illusory priors.

## Supplementary Material

Supplementary Material

## Data Availability

The data supporting the findings of this study are available from the corresponding author upon reasonable request. Due to ethical and privacy considerations, the data are not publicly available.
