# Supplementary material for: False but phonologically plausible linguistic priors induce cross-linguistic auditory illusions and attenuate electrophysiological markers of surprise
Source: Imaging Neurosci (Camb). 2026 Mar 26;4:IMAG.a.1178. doi: 10.1162/IMAG.a.1178 (PMC13023413; doi:10.1162/IMAG.a.1178)
Supplement: Supplementary Material [file IMAG.a.1178_supp.pdf]

# A Supplementary Materials

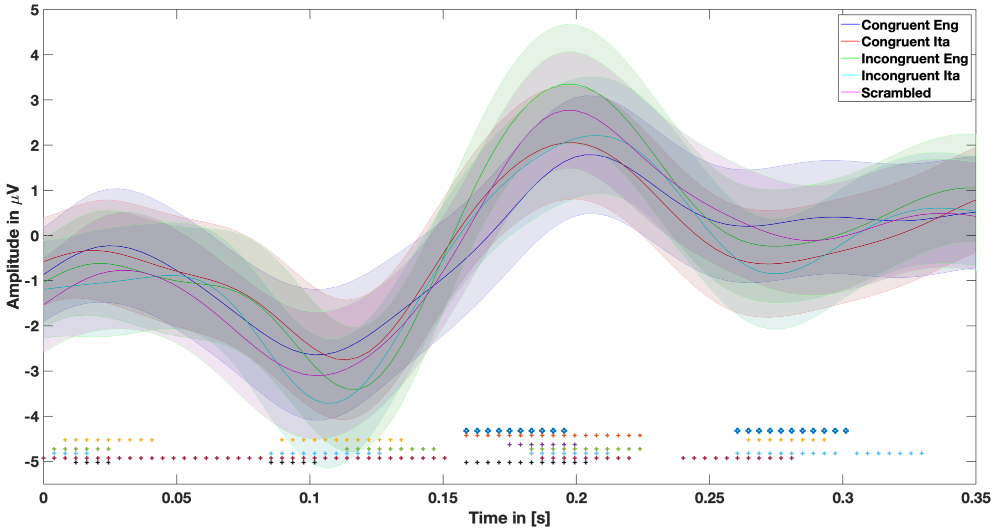


Figure S1: **Event-Related Potentials (ERPs) for the five experimental conditions.** Grand-average ERPs are displayed for the following conditions: **Congruent English (CEng)**, **Congruent Italian (CIta)**, **Incongruent English (IEng)**, **Incongruent Italian (IIta)**, and **Scrambled Words (SW)**. Data were bandpass filtered between 0.1 and 30 Hz and time-locked to the onset of the auditory stimulus (0 s). The ERP waveforms represent the mean amplitude (in $\mu$V) across a fronto-central electrode cluster over a 350 ms time window. Differences in waveform morphology and amplitude reflect distinct patterns of neural activity elicited by each linguistic condition. Colored dots along the x-axis indicate time points at which pairwise comparisons between conditions reached statistical significance ($p < 0.05$): **black** = CEng vs. CIta; **light blue** = CEng vs. IEng; **blue** = CEng vs. SW; **orange** = CIta vs. IEng; **red** = CIta vs. SW; **green** = IEng vs. IIta; **yellow** = IEng vs. SW; **purple** = IIta vs. SW.

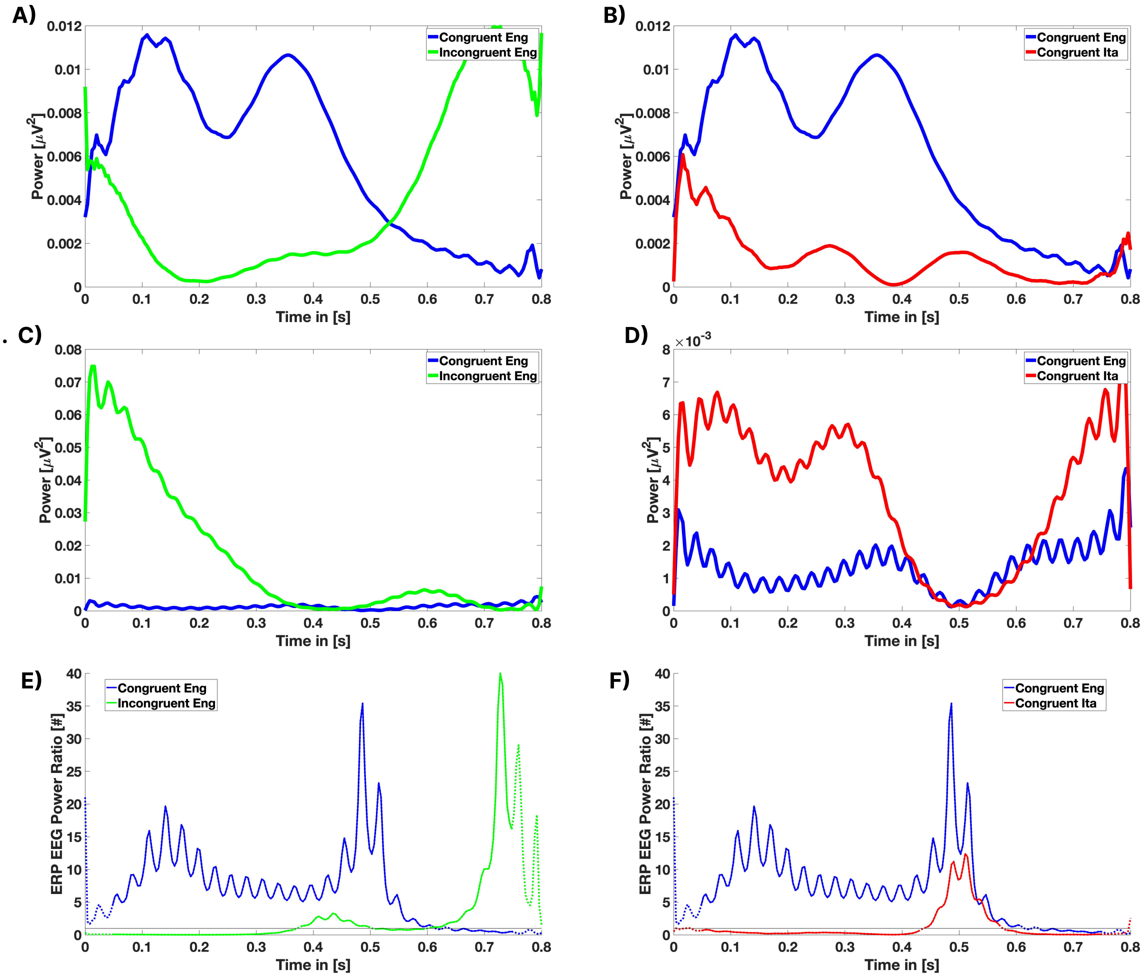


Figure S2: **Time-resolved EEG power and ERP power ratio across experimental conditions.** The figure shows both absolute EEG power (panels A–D) and ERP EEG power ratios (panels E–F) from a fronto-central electrode cluster, time-locked to auditory stimulus onset (0 s) over an 800 ms window. Power is expressed in $\mu V^2$ and reflects mean values across trials. **(A)** and **(B)**: Total power comparison between **Congruent English (CEng)** and either **Incongruent English (IEng)** (A) or **Congruent Italian (CIta)** (B), in the beta band (13–30 Hz). **(C)** and **(D)**: Power comparison in the gamma band (30–50 Hz) for CEng vs. IEng (C) and CEng vs. CIta (D). **(E)** and **(F)**: ERP EEG power ratio for the same CEng–IEgn and CEgn–CIta contrasts shown above, quantifying the relative strength of phase-locked (evoked) activity. These dynamics reveal distinct temporal and spectral patterns depending on the congruency and language of the prior context. In particular, CEng stimuli evoke stronger beta and gamma responses than incongruent or illusory conditions, especially between 100 and 500 ms.
